# Effects of Laser Therapy for Pain and Wound Recovery After Cesarean Section: A Systematic Review and Meta-Analysis of Randomized Controlled Trials

**DOI:** 10.3390/healthcare14213647

**Published:** 2026-10-28

**Authors:** Ahmed Abu-Zaid, Hajer G. H. Boresli, Latifah M. Madooh, Shaikha O. Alsadi, Dhoha M. Altamimi, Dema M. AlAnezi, Roua Y. Abdullah, Bibi Adel Bin Naser, Abdullah M. Alharran

**Affiliations:** 1 College of Medicine, Alfaisal University, Riyadh 11533, Saudi Arabia; 2 Al-Adan Hospital, Ministry of Health, Kuwait City 47005, Kuwait; 3 Farwaniya Hospital, Ministry of Health, Kuwait City 85000, Kuwait; 4 Al Sabah Area Maternity Hospital, Ministry of Health, Kuwait City 70000, Kuwait; 5 Faculty of Medicine, University of Jordan, Amman 11942, Jordan; 6 Royal College of Surgeons in Ireland, Busaiteen 228, Bahrain; 7 College of Medicine and Medical Sciences, Arabian Gulf University, Manama 26671, Bahrain

**Keywords:** cesarean section, photobiomodulation, scar, analgesic consumption, obstetrics, analgesia

## Abstract

Background: Evidence regarding laser therapy for acute incisional pain after cesarean section (CS) remains inconclusive, with individual trials reporting inconsistent findings. This study presents the first meta-analysis to systematically evaluate the efficacy and safety of laser therapy for postoperative pain following CS. Methods: Five databases were searched through July 2026 for randomized controlled trials (RCTs) evaluating laser therapy after CS. The primary outcome was pain intensity; secondary outcomes included time to first analgesic request, total analgesic consumption, scar outcomes, maternal prolactin level, and adverse events. Standardized mean differences (SMDs), mean differences (MDs), and risk ratios (RRs), with 95% confidence intervals (CIs), were pooled using random-effects models. Results: Eight RCTs involving 670 women were included. Laser therapy was associated with significant reduction in postoperative pain at 24 h (SMD: −0.95, 95% CI [−1.35, −0.55], *p* < 0.001, I^2^ = 44.27%) and at 48 h (SMD: −0.71, 95% CI [−1.01, −0.41], *p* < 0.001, I^2^ = 0%). Exploratory analyses suggested a longer time to first analgesic request (MD: 118.05 min, 95% CI [78.37, 157.74], *p* < 0.001, I^2^ = 97.10%) and lower total postoperative analgesic consumption (SMD: −2.18, 95% CI [−3.41, −0.96], *p* < 0.001, I^2^ = 93.83%) with laser therapy compared with control. However, each analysis included only two trials and demonstrated substantial heterogeneity, limiting confidence in these findings. There were no significant differences in Vancouver Scar Scale scores (*p* = 0.15), proportion of patients requesting any analgesia (*p* = 0.11), or adverse events (*p* = 0.45). Conclusions: This meta-analysis suggests that laser therapy may reduce acute incisional pain and analgesic requirements during the first 48 h after CS. However, substantial heterogeneity and limited evidence preclude conclusive recommendations. Larger, standardized RCTs are required to confirm efficacy, establish optimal treatment protocols, and clarify long-term benefits and safety.

## 1. Introduction

Cesarean section (CS) is one of the most common surgical procedures performed worldwide [1,2]. Although often necessary and lifesaving, CS is associated with postoperative morbidity [2]. Acute incisional pain is one of the most frequent and debilitating early complications after CS [3]. If not adequately managed, this pain can severely impair early mobilization, breastfeeding, maternal–infant bonding, sleep, self-care, and functional recovery [3]. Also, severe early postoperative pain may increase the risk of developing persistent postsurgical pain and lead to poorer long-term postpartum recovery [4,5].

The multimodal analgesia approach is the standard of care for post-CS pain management [6]. Commonly used pharmacological agents include acetaminophen, non-steroidal anti-inflammatory drugs (NSAIDs), and opioids when required for breakthrough pain [6,7]. Conventional approaches are generally effective but may be limited by side effects, specific contraindications, breastfeeding-related cautions, and limited healthcare access [8]. Hence, methods to reduce analgesia and opioid use are becoming more relevant in contemporary obstetrics, encouraging the investigation of safe, low-cost, and non-invasive adjuvant interventions [9].

Laser therapy is a non-thermal light therapy that has been investigated for its ability to modulate pain, reduce inflammation, and stimulate tissue repair [10]. Laser therapy can modulate inflammatory mediators, enhance local microcirculation, stimulate fibroblast activity to promote organized collagen synthesis, and decrease nociceptive signaling [11,12]. Connecting these effects to CS incision pain and wound healing makes laser therapy a plausible and promising approach to the postoperative care following CS. Several randomized controlled trials (RCTs) have evaluated laser therapy applied directly to the CS incision or the surrounding wound area [13,14,15,16,17,18,19,20]. However, while their findings are promising, they remain inconsistent, and differences in laser parameters, treatment protocols, and follow-up duration make the overall effect uncertain [13,14,15,16,17,18,19,20].

To our knowledge, no focused systematic review and meta-analysis has synthesized evidence of RCTs on laser therapy specifically after CS. Accordingly, this review aimed to evaluate the efficacy and safety of postoperative laser therapy for pain, analgesic requirements, and wound or scar recovery after CS.

## 2. Methods

### 2.1. Protocol Registration

Ethical approval was not required because this study involved only the analysis of previously published literature. The study design and reporting adhered to the Preferred Reporting Items for Systematic Reviews and Meta-Analyses (PRISMA) guidelines [21] and the Cochrane Handbook for Systematic Reviews of Interventions. This research was prospectively registered with the International Prospective Register of Systematic Reviews (PROSPERO) (identifier: CRD420261504418). Table S1 shows the PRISMA checklist.

### 2.2. Data Sources and Search Strategy

We performed a systematic literature search across five electronic databases (PubMed, Web of Science, CENTRAL, Scopus, and Embase) on 4 July 2026. The search strategy combined terms related to CS, cesarean wound or scar pain, and laser-based interventions. No restrictions based on country, publication year, or language were applied. The detailed search strategies and search outputs for each database are provided in Table S2. Additional searches included manual screening of the reference lists of eligible studies and targeted searches of Google Scholar to identify potentially missed trials.

### 2.3. Eligibility Criteria

We included RCTs based on the following PICO framework. The (P) population comprised postpartum women who underwent CS and received postoperative care for the cesarean wound. The (I) intervention was active non-ablative laser therapy applied to the recent cesarean incision or surrounding wound area, including low-level or high-power laser therapy, irrespective of wavelength, dose, energy density, number of treatment sessions, or timing of treatment initiation. The (C) comparators included sham or placebo laser, no laser therapy, standard postoperative care, or medical treatment alone. The primary (O) outcome was postoperative cesarean wound or incisional pain intensity measured 24 or 48 h after CS, irrespective of the pain assessment scale used. Pain outcomes assessed at other postoperative time points were also considered eligible but were summarized qualitatively when the timing of assessment was not sufficiently comparable for quantitative synthesis. Secondary (O) outcomes included requests for postoperative analgesia, time to first analgesic request, total postoperative analgesic consumption, wound or scar quality assessed using the Vancouver Scar Scale, quality of life or satisfaction outcomes when available, maternal prolactin levels, and adverse events.

We excluded non-randomized studies, observational studies, conference abstracts, protocols, reviews, editorials, animal studies, and studies without a direct eligible comparator. When trials included more than one eligible active laser arm, these arms were combined into a single laser therapy group when clinically appropriate. Similarly, when trials included more than one eligible control arm, such as sham laser and no-laser controls, the control arms were combined to yield a single independent comparison.

### 2.4. Study Selection

Study selection was conducted using Covidence software (Veritas Health Innovation, Melbourne, Australia). Following duplicate removal, at least two independent reviewers screened records in two stages: first by title and abstract, and then by full text for potentially eligible studies. Disagreements regarding eligibility were resolved through discussion and consensus among the reviewers.

### 2.5. Data Extraction

A standardized Excel extraction form was developed and pilot-tested using a subset of eligible studies. Extracted data were grouped into three main categories. First, the study characteristics included author, year, country, study design, trial setting, total sample size, CS type or timing after CS at enrolment, laser device and modality, wavelength, power, energy density or dose, treatment site, number and timing of sessions, co-interventions, control intervention, primary outcome, and follow-up duration. Second, the baseline participant characteristics included age, gestational age, body mass index, gravidity, parity, diabetes status, smoking status, and baseline wound or pain status when reported. Third, the outcome data included all primary and secondary outcomes described above, including postoperative pain scores, analgesia request, time to first analgesic request, total analgesic consumption, Vancouver Scar Scale, quality-of-life or satisfaction outcomes, and adverse events.

Data extraction was performed independently by at least two reviewers, and discrepancies were resolved by discussion or consultation with a third reviewer. Dichotomous outcomes were extracted as the number of events and the total number of randomized or analyzed participants. Continuous outcomes were extracted as means and standard deviations (SDs). When means and SDs were not directly reported, standard conversion methods were applied. Outcomes reported as medians and interquartile ranges were converted to means and SDs using the methods of Wan et al. [22]. When studies reported 95% confidence intervals (CIs) around a mean without SDs, SDs were calculated algebraically from the confidence interval limits according to the Cochrane Handbook for Systematic Reviews of Interventions [23].

### 2.6. Risk of Bias and Certainty of Evidence

The risk of bias for each included RCT was independently assessed by at least two reviewers using the revised Cochrane Risk of Bias 2 (RoB 2) tool [24]. The assessment considered bias arising from the randomization process, deviations from intended interventions, missing outcome data, measurement of the outcome, and selection of the reported result. Disagreements were resolved through discussion and consensus.

The certainty of evidence for each major outcome was evaluated using the Grading of Recommendations Assessment, Development and Evaluation (GRADE) framework [25]. The certainty of evidence was rated across the domains of risk of bias, inconsistency, indirectness, imprecision, and publication bias. All judgments were justified and documented. Any discrepancies were resolved by discussion or consultation with a third reviewer.

### 2.7. Statistical Analysis

All statistical analyses were performed using Stata/MP software, version 19 (StataCorp LLC, College Station, TX, USA), for Windows. Dichotomous outcomes were analyzed using risk ratios (RRs). For dichotomous outcomes with zero events, a continuity correction of 0.5 was applied. Continuous outcomes were analyzed using mean differences (MDs) when outcomes were reported on the same scale. Hedges’ g standardized mean differences (SMDs) were used when clinically similar continuous outcomes were reported using different scales. All effect estimates were reported with 95% CIs. For postoperative pain, quantitative syntheses were performed separately for assessments at 24 and 48 h after CS. Pain measurements obtained at other postoperative time points were not pooled when assessment timing differed substantially among studies and were instead summarized qualitatively. This approach was used to avoid combining pain outcomes measured at clinically distinct stages of postoperative recovery. Random-effects models using restricted maximum likelihood estimation (REML) were applied as the primary analytical approach to account for expected clinical and methodological heterogeneity across the included trials. Statistical heterogeneity was assessed using the Chi-squared test and quantified using the I^2^ statistic. Substantial heterogeneity was defined as a Chi-squared *p*-value < 0.10 or an I^2^ value > 50%.

When sufficient data were available, subgroup analyses were conducted by laser type and by the timing of treatment initiation after CS. Tests for subgroup differences were used to assess whether the pooled effect differed across prespecified subgroups. In cases of substantial heterogeneity, leave-one-out sensitivity analysis was conducted to explore the influence of individual studies on the pooled estimate. Galbraith plots were also used, when applicable, to visually assess the contribution of individual studies to between-study heterogeneity. Publication bias was not assessed because all meta-analyzed outcomes included fewer than 10 trials [26].

## 3. Results

### 3.1. Search Results and Study Selection

**Figure 1:**
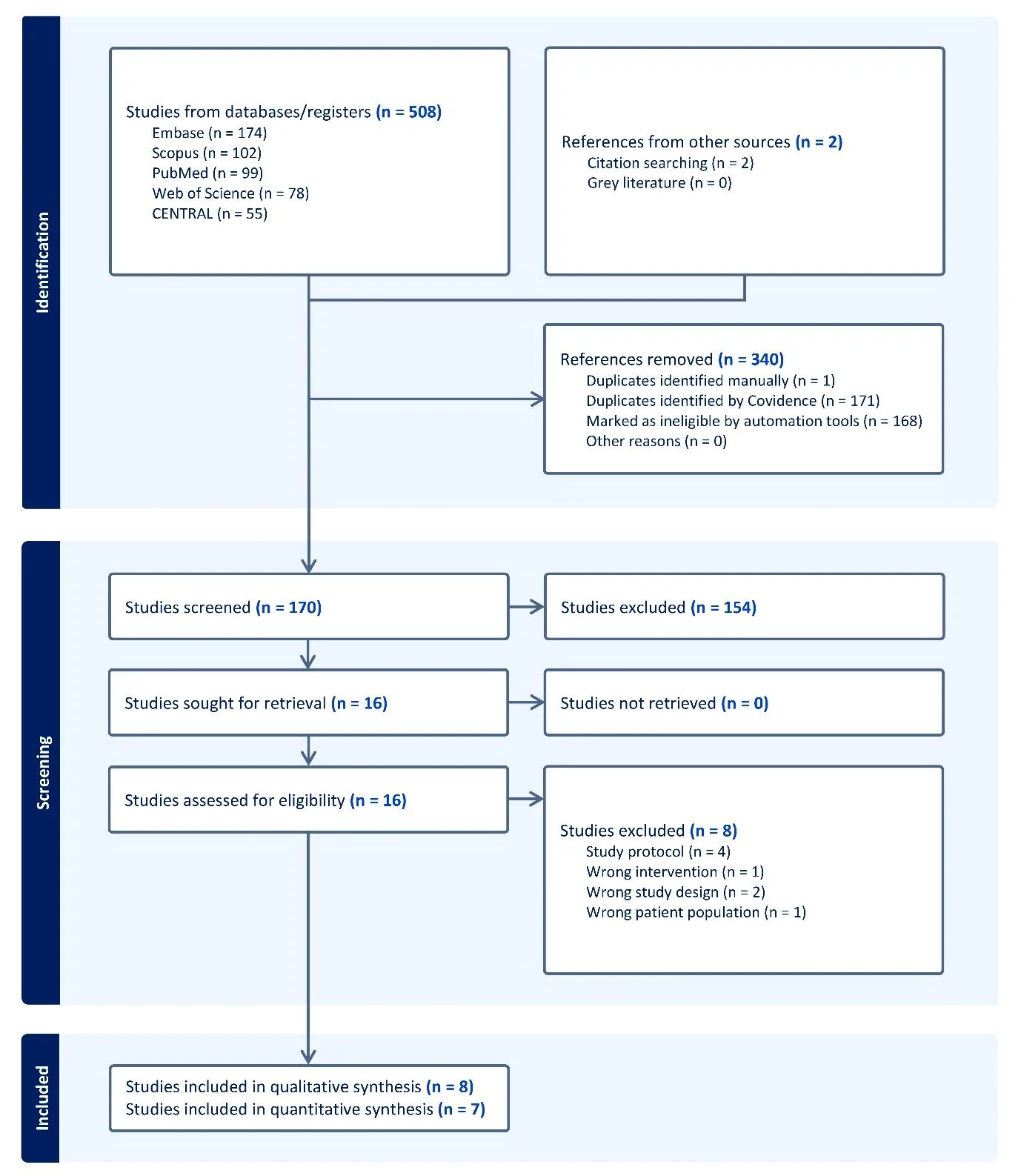
PRISMA flow chart of the study screening and selection process.

A total of 510 records were identified through the literature search, including 508 retrieved from five databases and two identified through citation searching. Before screening, 340 references were removed, including 172 duplicates and 168 records marked as ineligible by automation tools. The 168 records were classified as ineligible using Covidence’s built-in machine-learning-based automated screening function. This automated classification was used to facilitate screening, and the screening process was subsequently verified by the review team; no potentially eligible study was identified among the records excluded by automation. The remaining 170 records were screened, of which 154 were excluded after failing to meet the inclusion criteria. Sixteen full-text reports were assessed for eligibility, and eight were excluded for various reasons (Table S3). Finally, eight trials were included in the qualitative synthesis [13,14,15,16,17,18,19,20], of which six were included in the quantitative synthesis [14,15,16,17,19,20] (Figure 1).

### 3.2. Characteristics of Included Studies

Eight RCTs, involving 670 patients, were included in this review [13,14,15,16,17,18,19,20]. Four trials were conducted in Iran, two in Brazil, and two in Egypt. All trials enrolled postpartum women after elective CS. A key characteristic of the included evidence was the difference in the laser modality and application protocol, ranging from low-level laser therapy using red or infrared wavelengths to high-level laser therapy. Laser was applied either immediately after wound closure or during the early postoperative period, with treatment schedules ranging from a single session to repeated sessions over several days or weeks. Comparator arms involved sham laser, placebo/off-laser application, or standard postoperative care. Follow-up duration ranged from early postoperative assessments within 24–48 h to longer wound and scar evaluations extending up to six months. Detailed study designs and baseline participant characteristics are summarized in Table 1 and Table 2, respectively. In the study by Kahkhaie et al. [17], laser power density was inconsistently reported as 2 W/cm^2^ in the Methods section and 4 W/cm^2^ in the Abstract, Discussion, and Conclusion. We extracted 4 W/cm^2^ as the more consistently reported value, although this discrepancy could not be definitively resolved.

### 3.3. Summary of Risk of Bias and Certainty of Evidence

**Figure 2:**
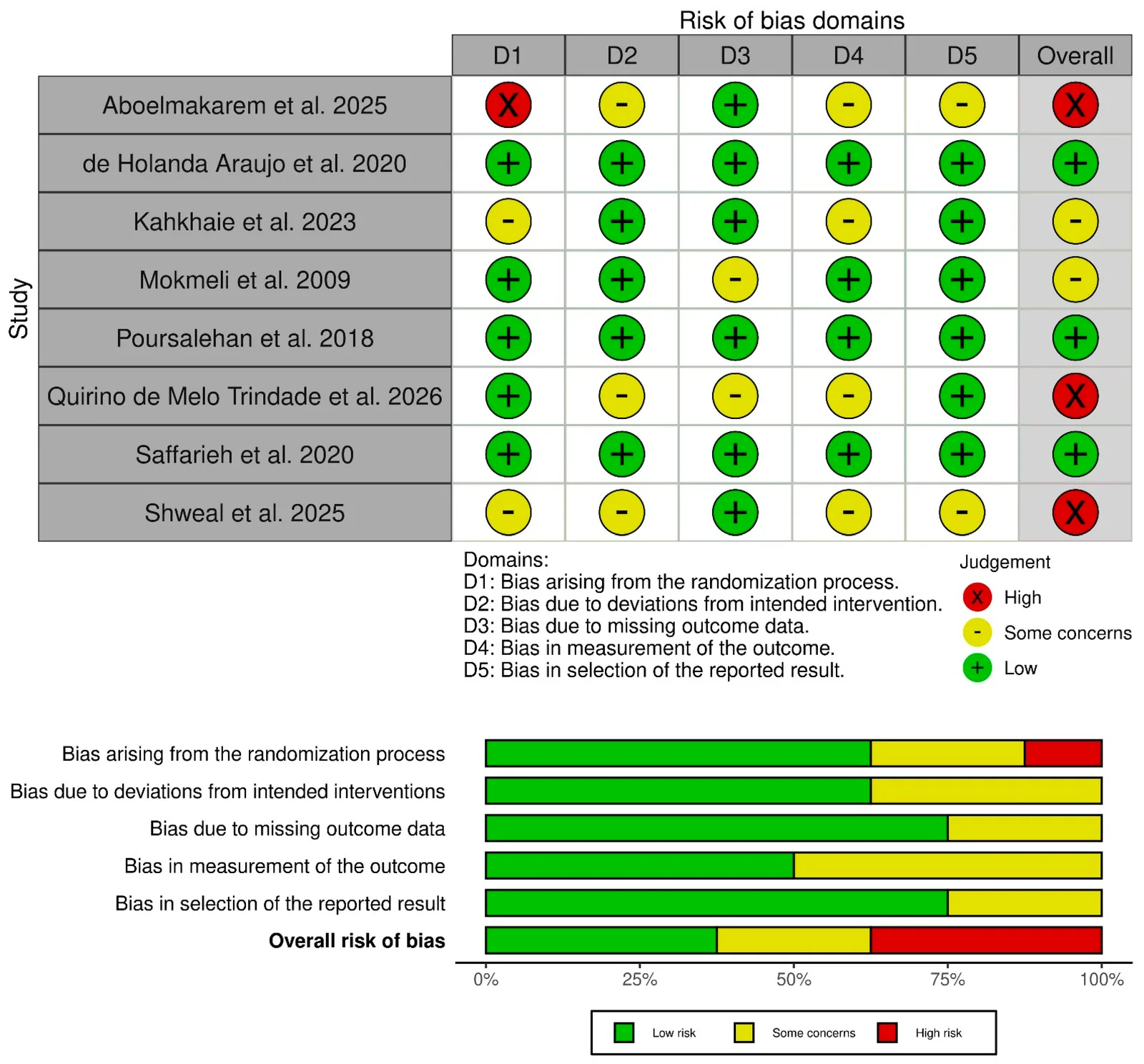
Quality assessment of risk of bias in the included RCTs using the RoB 2 tool [13,14,15,16,17,18,19,20].

Among the included RCTs, three trials were judged to have a low overall risk of bias [14,19,20], two trials raised some concerns [17,18], and three trials showed a high overall risk of bias [13,15,16] (Figure 2).

The certainty of evidence ranged from moderate to very low. Moderate-certainty evidence supported reductions in pain at 24 and 48 h, while evidence for time to first analgesic request, total analgesic consumption, scar outcomes, and adverse events was very low. Evidence for any analgesia request was rated low. Overall, confidence in most estimates was limited by risk of bias, heterogeneity, and imprecision, warranting cautious interpretation. The detailed GRADE certainty of evidence assessment is outlined in Table 3.

### 3.4. Primary Outcome: Pain

**Figure 3:**
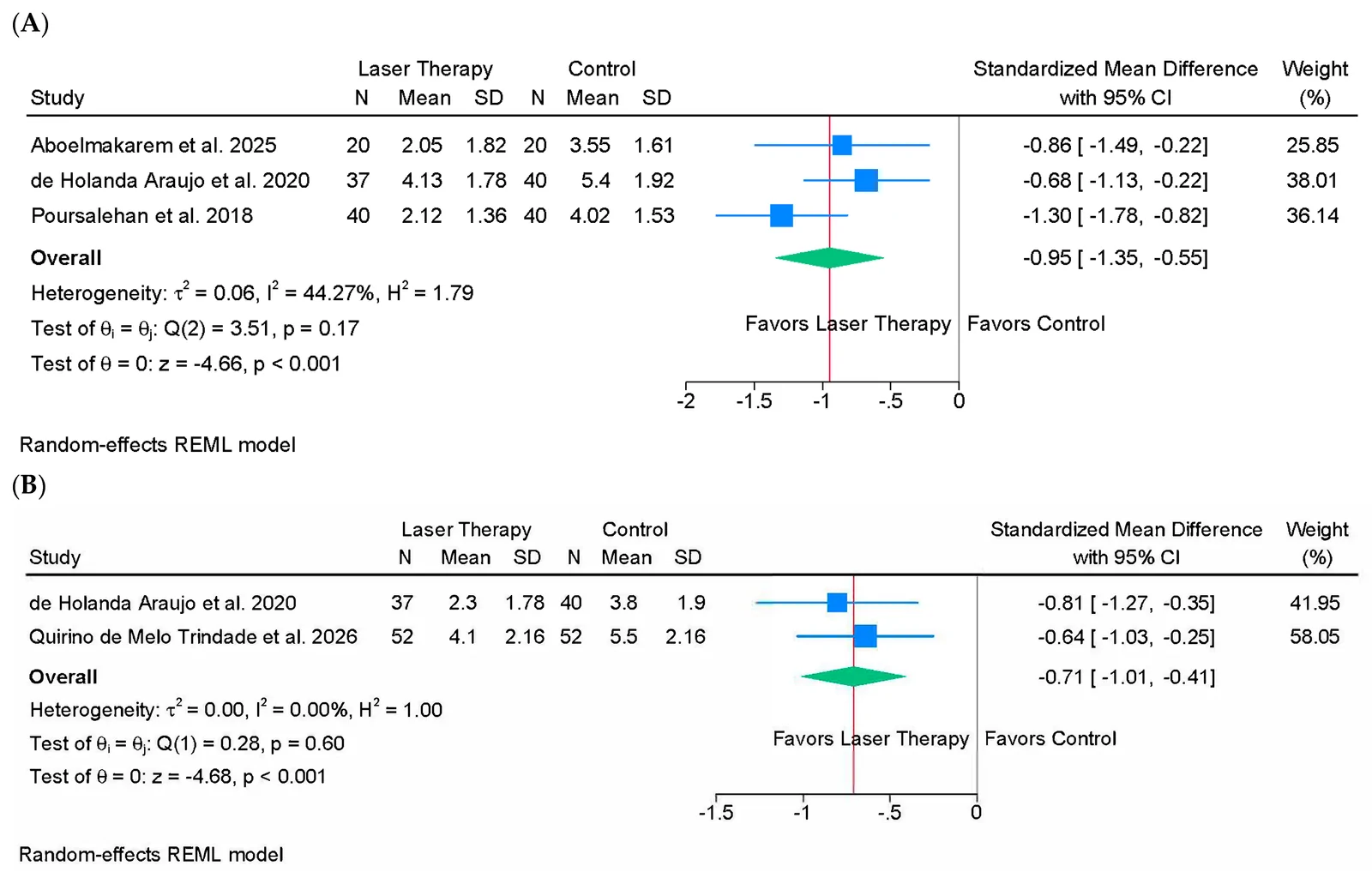
Forest plots comparing laser therapy with control for postoperative pain outcomes at (**A**) 24 h and (**B**) 48 h [15,16,19,20].

Laser therapy was associated with significant reduction in postoperative pain at 24 h (SMD: −0.95, 95% CI [−1.35, −0.55], *p* < 0.001, I^2^ = 44.27%) (Figure 3A) and at 48 h (SMD: −0.71, 95% CI [−1.01, −0.41], *p* < 0.001, I^2^ = 0%) (Figure 3B). Other studies assessed postoperative pain at different time points and were therefore not quantitatively pooled. Kahkhaie et al. [17] assessed pain using a 10-point VAS ranging from 1 to 10 and reported significantly lower pain scores in the laser group than in the control group on postoperative day 3 (5.15 ± 1.67 vs. 9.17 ± 1.31), day 7 (2.26 ± 1.46 vs. 5.97 ± 1.24), day 14 (1.36 ± 1.17 vs. 4.01 ± 1.41), and 6 weeks after surgery (0.44 ± 0.62 vs. 2.25 ± 1.07; all *p* < 0.001). Saffarieh et al. [14] assessed pain using an 11-point VAS ranging from 0 (no pain) to 10 (severe pain) and reported significantly lower pain scores in the laser group than in the control group on postoperative day 5 (4.15 ± 0.84 vs. 5.61 ± 1.33) and day 10 (1.43 ± 0.66 vs. 2.51 ± 1.00; all *p* < 0.0001). Shweal et al. [13] also assessed postoperative pain using a 10-point VAS and reported lower pain scores in the laser group than in the control group (2.36 ± 0.70 vs. 5.89 ± 0.82, *p* = 0.001); however, the timing of pain assessment was not clearly specified, but likely postoperative day 4.

### 3.5. Secondary Outcomes

#### 3.5.1. Analgesia Requirement

**Figure 4:**
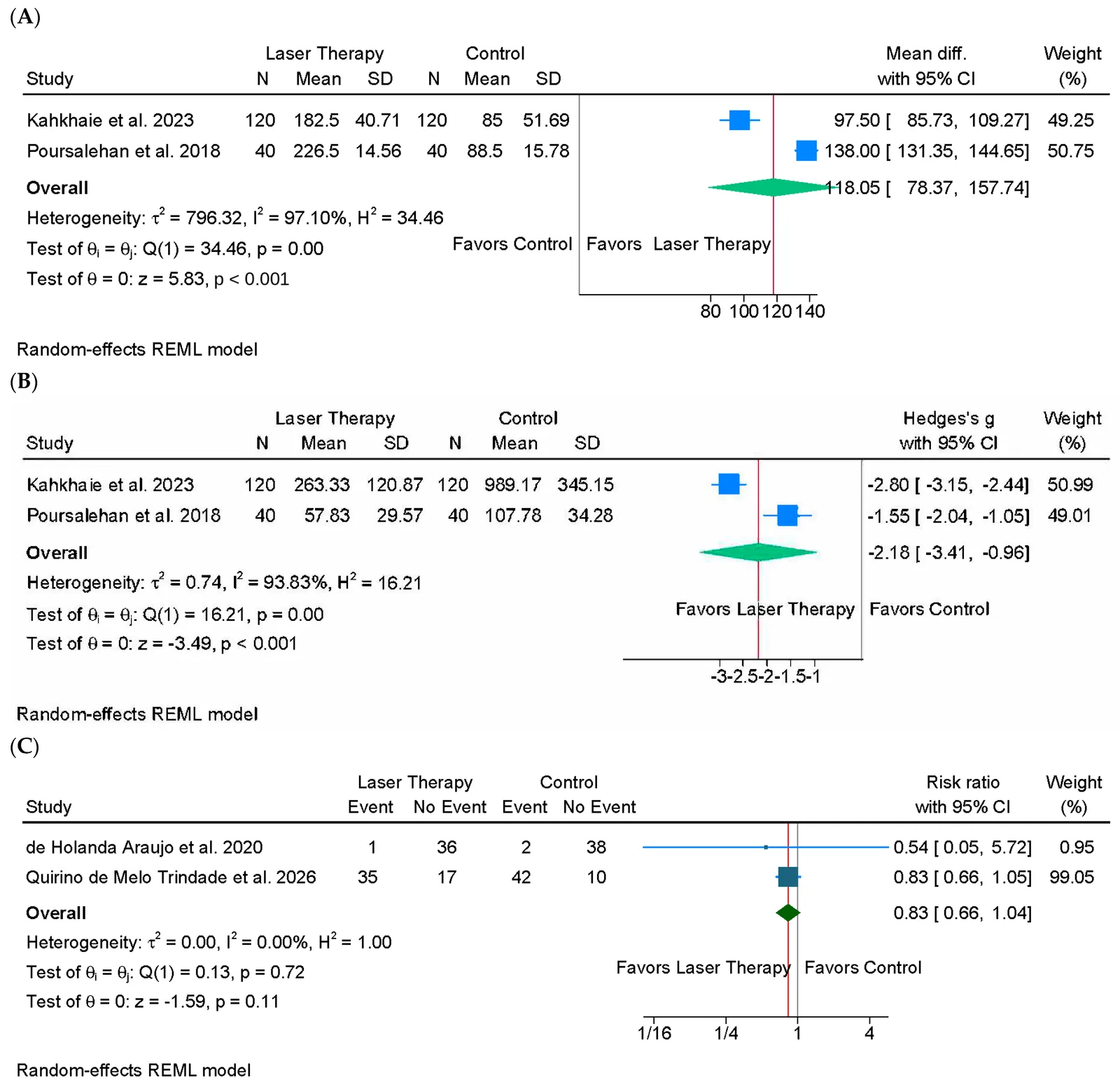

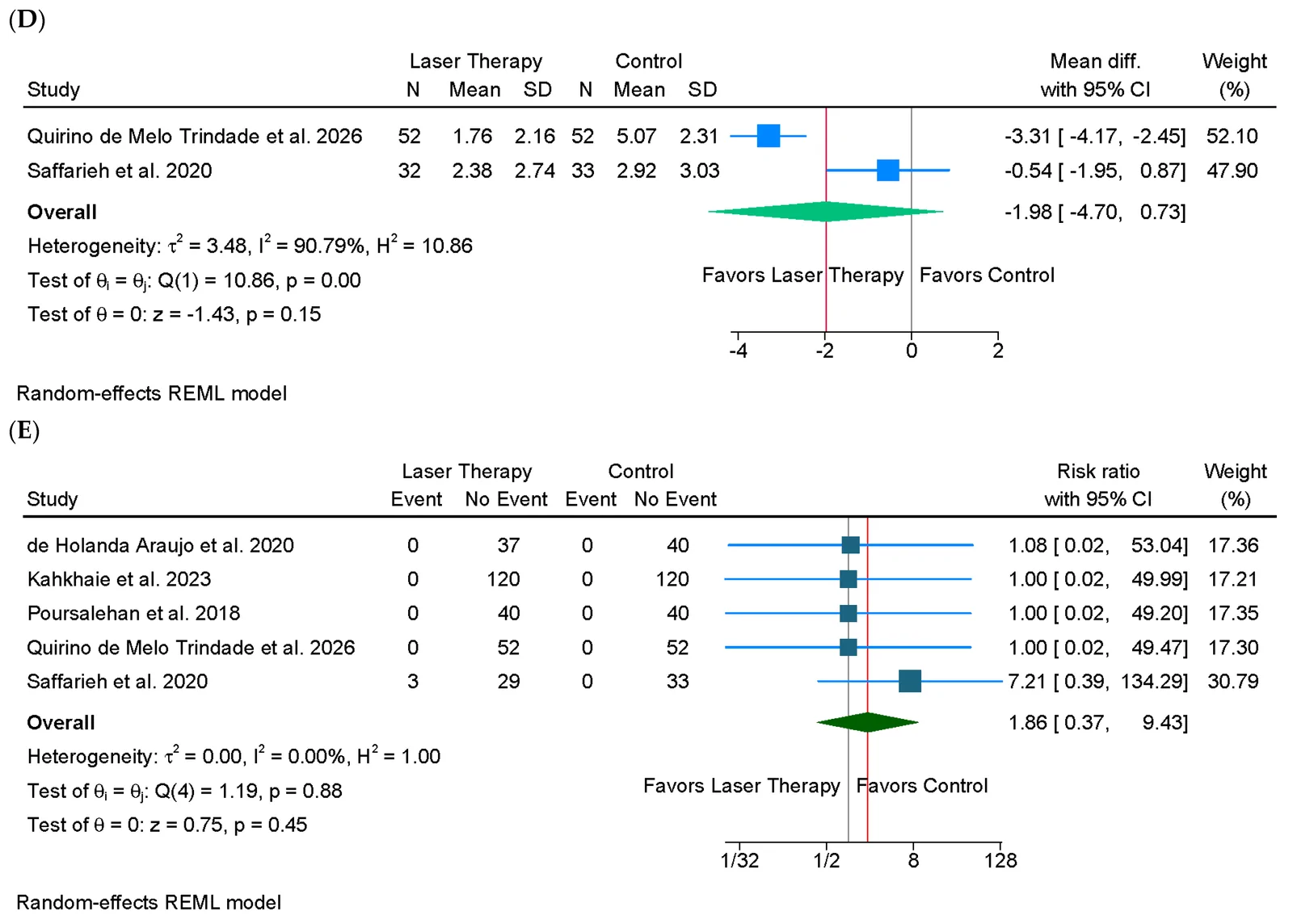
Forest plots comparing laser therapy with control for secondary efficacy and safety outcomes: (**A**) mean time to first analgesic request, (**B**) mean total postoperative analgesic dose, (**C**) rate of any analgesic request, (**D**) mean Vancouver Scar Scale score, and (**E**) rate of adverse events [14,15,17,19,20].

Exploratory analyses suggested that laser therapy may prolong the time to first analgesic request compared with control (MD: 118.05 min, 95% CI [78.37, 157.74], *p* < 0.001, I^2^ = 97.10%) (Figure 4A) and reduce total postoperative analgesic consumption (SMD: −2.18, 95% CI [−3.41, −0.96], *p* < 0.001, I^2^ = 93.83%) (Figure 4B). However, both analyses included only two trials and demonstrated substantial heterogeneity, warranting cautious interpretation. However, there was no significant difference between both groups in the proportion of patients requesting any analgesia (RR: 0.83, 95% CI [0.66, 1.04], *p* = 0.11, I^2^ = 0%) (Figure 4C).

#### 3.5.2. Wound Healing and Scar Characteristics

There was no significant difference between both groups in the Vancouver scar scale (MD: −1.98, 95% CI [−4.70, 0.73], *p* = 0.15, I^2^ = 90.79%) (Figure 4D). Also, Quirino de Melo Trindade et al. reported domain-specific assessment, showing improvements in vascularity and pliability, with no significant differences in scar thickness or pigmentation [15]. In contrast, Saffarieh et al. found no significant differences between groups in vascularity, pigmentation, pliability, and height domains [14]. Additionally, Kahkhaie et al. reported a significantly shorter time to suture removal in the laser group (*p* < 0.001) [17].

#### 3.5.3. Safety

There was no significant difference between laser therapy and control in adverse events (RR: 1.86, 95% CI [0.37, 9.43], *p* = 0.45, I^2^ = 0%) (Figure 4E). Saffarieh et al. reported itching in three women receiving laser therapy compared with none in the control group [14].

#### 3.5.4. Maternal and Endocrine Outcomes

Mokmeli et al. investigated the effects of combined local and intravenous low-level laser therapy on maternal prolactin secretion and lactation. On postoperative day 3, mean serum prolactin was numerically higher in the laser group than in the control group (333.57 ± 103.40 vs. 303.00 ± 71.06 µg/L); however, this difference was not statistically significant (*p* = 0.205) [18]. Also, Shweal et al. assessed serum cortisol as an exploratory biomarker of the postoperative stress response. Serum cortisol decreased significantly from baseline in both groups, but the post-treatment level was significantly lower in the laser group than in the control group [13].

## 4. Discussion

### 4.1. Summary of Principal Findings

This systematic review and meta-analysis synthesized eight RCTs and 670 patients evaluating laser therapy after CS. Laser therapy was associated with lower postoperative pain at 24 and 48 h. Other studies also reported significant reductions in postoperative pain at different time points, although the heterogeneity in assessment timing precluded quantitative pooling. It also prolonged the time to the first analgesic request and reduced total postoperative analgesic consumption. Still, laser therapy did not significantly reduce the proportion of women who requested any analgesia, improve the pooled Vancouver Scar Scale score, or increase the risk of adverse events, though these findings should be interpreted cautiously. The certainty of these findings varied: evidence for pain at 24 and 48 h was graded as moderate, whereas evidence for time to first analgesia, total analgesic dose, scar quality, and adverse events was low or very low.

### 4.2. Interpretation of Findings

The reductions in pain at 24 h and at 48 h are clinically relevant because the first two postoperative days are often the most painful period of maternal recovery. During this interval, women are expected to start breastfeeding, care for their newborn, and regain a degree of independence despite having undergone major abdominal surgery. Poorly controlled pain can interfere with each of these activities and is associated with impaired mobility, sleep, mood, and functional recovery [27,28]. Observational evidence has also shown that severe acute postpartum pain is associated with subsequent persistent pain [28,29]. Accordingly, the consistent alleviation of pain with laser therapy after 24 and 48 h is encouraging.

The standardized effects also require careful clinical interpretation. Although the pooled effects at 24 and 48 h were statistically significant, statistical significance does not necessarily indicate a clinically important reduction in pain. SMDs do not directly represent change on a familiar pain scale and may be influenced by differences in outcome measurement and variability across studies [30]. Where individual trials reported pain outcomes on interpretable scales, the magnitude of the between-group differences should be considered in relation to commonly used thresholds for clinically meaningful pain improvement. However, differences in pain scales, baseline pain severity, assessment timing, and reporting methods limit the application of a single minimal clinically important difference across all included trials [31,32]. Accordingly, the present findings provide evidence of a reduction in postoperative pain but do not establish that the magnitude of benefit consistently exceeded a patient-important threshold in all studies. Future trials should report absolute between-group differences alongside standardized effect estimates and the proportion of participants achieving clinically meaningful pain reduction.

Furthermore, laser therapy prolonged the time to first analgesic request by approximately 118 min and reduced total postoperative analgesic dose. These outcomes suggest that the intervention may not only change pain ratings but also influence medication requirements. Such an effect would be clinically attractive within contemporary enhanced-recovery pathways, which prioritize scheduled non-opioid analgesics, limited rescue opioids, early mobilization, and preservation of maternal alertness and function [6,33,34]. Concerns about unnecessary opioid exposure and unused postdischarge medication have further strengthened interest in opioid-sparing strategies after cesarean delivery [35,36].

However, only two trials contributed to each of the time-to-analgesia and total-dose analyses, and heterogeneity exceeded 90%. The studies differed in rescue thresholds, background analgesics, dose measurement, and treatment schedules [17,19]. The large standardized reduction in total analgesic dose may therefore reflect benefit, but it may also partly reflect differences in how analgesia was administered and recorded. The lack of a significant reduction in analgesic requests reinforces this uncertainty. Laser therapy, therefore, could delay the first request or reduce repeated dosing without preventing women from requesting at least one analgesic, as a binary endpoint ignores information about timing, frequency, and cumulative exposure and may be less sensitive than total consumption. Still, because each analysis was based on only two trials and heterogeneity exceeded 90%, these findings should be considered exploratory and hypothesis-generating rather than evidence of an established analgesic-sparing effect. The observed prolongation of time to first analgesic request and reduction in cumulative analgesic consumption warrant confirmation in adequately powered trials using standardized background analgesia, rescue criteria, and methods of reporting analgesic consumption.

Moreover, evidence for wound and scar recovery was less convincing than evidence for pain. The pooled Vancouver Scar Scale score did not differ significantly between the laser therapy and control groups, and the included trials yielded conflicting results for its subscales. This may be because scar remodeling continues for months, and assessment at 48 h largely reflects acute wound inflammation rather than the scar’s final appearance or mechanical behavior. The Vancouver Scar Scale was originally developed for burn scars and evaluates pigmentation, vascularity, pliability, and height [37]. However, it is commonly used in surgical research; still, it does not fully capture patient-reported symptoms, cosmetic satisfaction, pain, or functional restriction [38,39]. Moreover, assessments performed within 24–48 h after cesarean section primarily reflect early wound healing rather than definitive scar formation or remodeling. Scar characteristics evolve over substantially longer periods, and early VSS scores may therefore have limited relevance to long-term scar outcomes. Future trials should incorporate validated patient-reported scar assessment tools alongside clinician-assessed measures and extend follow-up sufficiently to capture the scar remodeling phase. Reviews of scar-measurement instruments have indicated variability in reliability and responsiveness, especially when scales are applied outside their original setting [38,39]. Earlier research has suggested that laser therapy may promote aspects of tissue repair, but they have also shown significant variation across wound types and doses [40,41,42,43]. Similarly, trials of photobiomodulation for perineal pain and episiotomy healing have produced mixed results, with some reporting little or no improvement despite plausible biological mechanisms [44,45,46]. Accordingly, the current evidence supports an analgesic effect more clearly than a scar-modifying effect.

The findings on wound and scar recovery should also be considered within the broader literature on adjunctive regenerative approaches after cesarean section. Recent evidence on platelet-rich plasma (PRP) provides complementary context, with a 2026 systematic review and meta-analysis of five RCTs reporting improvements in patient- and clinician-assessed scar quality and early wound inflammation, although early postoperative pain was not significantly reduced [47]. A 2025 randomized pilot trial similarly reported favorable scar outcomes with intraoperative PRP, particularly at 40 days [48]. These findings suggest that regenerative strategies may have potential for improving post-cesarean wound and scar healing, but the available evidence remains limited and heterogeneous. Importantly, this literature does not provide evidence regarding the efficacy of laser therapy itself; rather, it highlights the need for longer follow-up and standardized assessment when evaluating interventions targeting cesarean wound and scar recovery.

### 4.3. Strengths and Limitations

This review has several strengths: it focused specifically on randomized evidence, searched multiple databases and other sources, evaluated time-specific pain outcomes, and incorporated both efficacy and safety endpoints. Risk of bias was assessed using RoB 2, and the certainty of evidence was formally rated using GRADE. Random-effects models were appropriate given the expected clinical diversity, while leave-one-out, Galbraith, and subgroup analyses helped explore the high heterogeneity.

Nevertheless, our results remain limited by the following: first, only eight trials were eligible; some outcomes were based on two studies; and five trials were judged to have either some concerns or a high overall risk of bias. Second, pain was particularly vulnerable to performance and detection bias when sham procedures and blinding could be inadequate. Third, treatment protocols varied, and the reporting of laser parameters was sometimes incomplete or inconsistent. Specifically, wavelength, power, energy density, treatment frequency, and timing differed across trials, which may have contributed to clinical heterogeneity and limited the extent to which the pooled effects can be attributed to a specific laser regimen. Fourth, pain outcomes were assessed at different postoperative time points across trials. To address this clinical heterogeneity, we restricted quantitative synthesis of pain to the more comparable 24- and 48-h assessments and summarized outcomes at other time points qualitatively. Fifth, only two trials contributed to each of the analyses of time to first analgesic request and total analgesic consumption, and both analyses demonstrated substantial heterogeneity, limiting the precision and interpretability of these findings. In addition, background analgesic regimens, rescue thresholds, and methods of measuring total analgesic consumption were not standardized across trials. Sixth, included trials were conducted in a limited number of countries and largely enrolled women undergoing elective CS under spinal anesthesia, which may restrict the generalizability of our findings to emergency procedures, general anesthesia, complicated pregnancies, or women with comorbidities. Finally, publication bias could not be formally assessed because fewer than ten studies contributed to each meta-analysis.

### 4.4. Implications for Clinical Practice and Future Research

Clinically, laser therapy can be viewed as a potential adjunct to, rather than a substitute for, established multimodal analgesia. Current recommendations support neuraxial opioids where appropriate, scheduled acetaminophen and non-steroidal anti-inflammatory drugs, and rescue opioids for breakthrough pain, alongside early feeding, mobilization, breastfeeding support, and patient-centered discharge planning [6,33,34]. A non-invasive intervention that reduces pain without causing sedation or systemic drug exposure would be attractive when medication options are limited by intolerance or contraindications. Still, implementation would require access to a suitable device, trained personnel, protective equipment, infection-control procedures, and time for repeated treatments. The intervention may be more feasible when a short, standardized protocol is shown to work than when several sessions over multiple days are required. No economic analyses were available to determine whether equipment and staffing costs can be offset by lower medication use, shorter recovery, or improved patient experience.

Future trials should be multicenter, adequately powered, and sham-controlled whenever possible. The intervention itself should be described using established laser-reporting recommendations and the TIDieR checklist so that effective protocols can be reproduced rather than approximated [49]. Trials should use common assessment time points (6, 12, 24, and 48 h) and report pain at rest and during movement. Analgesic use should be presented by drug class, timing, number of rescue doses, and, when appropriate, standardized equivalent dose. Finally, direct comparisons of a standardized low-level protocol with a standardized high-power protocol may clarify whether the laser power provides additional benefit.

## 5. Conclusions

Laser therapy appears to reduce early pain after CS and may delay the first analgesic request and decrease cumulative analgesic use. The most consistent quantitative evidence was observed at 24 and 48 h, while pain outcomes assessed at other postoperative time points were heterogeneous and were therefore summarized qualitatively rather than pooled. The evidence is most promising for pain during the first 48 h, whereas the optimal treatment protocol and any benefit for wound or scar recovery remain uncertain. The findings for time to first analgesic request and cumulative analgesic consumption were based on only two trials with substantial heterogeneity and should therefore be considered exploratory and hypothesis-generating rather than evidence of an established analgesic-sparing effect. Given the low or very low certainty of evidence for several outcomes and the risk of bias in some included trials, these findings should be interpreted cautiously. No clear safety signal was detected, but the available trials were too small to exclude uncommon harm. Laser therapy should therefore remain an adjunctive intervention to, rather than a replacement for, standard multimodal analgesia until larger, methodologically rigorous trials establish a reproducible protocol and clarify its clinically meaningful benefits and longer-term effects on wound and scar recovery.

## Figures and Tables

**Table 1:** Summary characteristics of the included RCTs.

Study ID	Country	*n*	Laser Intervention Details	Control Details	Co-Interventions	Treatment Schedule	CS Type/Timing After CS at Enrollment	Main Inclusion Criteria	Primary Outcome	Follow-Up Duration
Aboelmakarem et al. 2025 [16]	Egypt	40	HLLT using LUMIX^®^ 2 double diode device; 904 nm; frequency 6 kHz; duty cycle 100%; applied punctually/non-contact along CS suture, 1-cm spacing between points; 10 s/point	Ketoprofen injection only	Ketoprofen injection in both groups	Started 24 h postpartum; 3 times/day every 8 h for 7 days	Elective CS; intervention started 24 h after CS	Females aged 20–35 years, BMI < 32 kg/m^2^, primiparous, full-term pregnancy, scheduled for elective CS	Postoperative pain by VAS	Pain was assessed at 1, 4, 8, 12, 16, and 24 h after treatment; the intervention course was 7 days
de Holanda Araujo et al. 2020 [20]	Brazil	77	LLLT with GaAlAs Laserpulse IBRAMED^®^; continuous 660 nm, 30 mW; Experimental I: 4 J/cm^2^, 0.24 J/point, 8 s/point, total energy 4.8 J; Experimental II: 2 J/cm^2^, 0.12 J/point, 4 s/point, total energy 2.4 J; 10 points, 1-cm spacing along incision	Two controls: no LLLT control group and placebo laser with device on, but no energy emission	Pharmacological analgesia, including tenoxicam, scopolamine, dipyrone, and simethicone	Two sessions at 12 h and 20–24 h postpartum	Immediate postpartum after CS; first assessment 12 h after CS	Women > 18 years, immediate postpartum after CS, no clinical/obstetric intercurrences such as hypertension or diabetes, mean NRS pain ≥ 3	Pain by NRS and algometry	Final assessment at 44–48 h postpartum
Kahkhaie et al. 2023 [17]	Iran	240	LLLT with cluster current; wavelength 810 nm; power 300 mW; reported power density 2 W/cm^2^ in methods and 4 W/cm^2^ in abstract/discussion/conclusion; applied to surgical incision	Placebo	Routine antibiotics and painkillers	NR	Selective/elective CS under spinal anesthesia; randomization after operation	Pregnant women referred for selective CS	NR	Up to 6 weeks after surgery; pain reported on days 3, 7, 14, and 6 weeks
Mokmeli et al. 2009 [18]	Iran	14	Combined local and intravenous LLLT: GaAlAs 980 nm, 100 mW, 3.3 J/cm^2^, 2 J/point, total 60 J over incision/umbilicus-to-incision line; GaAlInP 650 nm, 30 mW, 1.5 J/cm^2^, 0.9 J/point, total 27 J; matrix probe 860/650 nm over surrounding area; plus IV 650 nm laser, 2.5 mW for 15 min	Placebo	Routine wound care and identical environmental factors	Three sessions on three consecutive postoperative days; first session soon after surgery in recovery room, second and third on postoperative days 2 and 3	Scheduled CS; intervention began soon after surgery	Normal healthy women scheduled for CS	Blood prolactin/lactation safety	Prolactin measured on postoperative day 3; wound-margin tissue sample on postoperative day 10
Poursalehan et al. 2018 [19]	Iran	80	LLLT using combined IR and red lasers: PLP-IR 808 nm, 200 mW, and PLP-R 650 nm, 100 mW; incision treated with red 1 J/cm^2^ for 10 s + IR 2 J/cm^2^ for 10 s, total 3 J/cm^2^ on suture; surrounding tissue treated at 6 points, total 4.5 J/cm^2^/point	Placebo	Spinal anesthesia with bupivacaine; intravenous Pethidine for pain (VAS ≥ 3)	Single intraoperative/immediate postoperative application at the end of surgery, before bandage	Elective CS using spinal anesthesia and Pfannenstiel incision	Pregnant women aged 18–40 years, first or second pregnancy, ASA I–II, candidates for elective CS	Postoperative pain by VAS; first analgesic request and total analgesic consumption	24 h after surgery; VAS at 1, 4, 8, 12, 16, and 24 h
Quirino de Melo Trindade et al. 2026 [15]	Brazil	104	LLLT with diode laser; Laser Power Therapy; ECCO^®^ Reability; continuous mode; wavelength stated as 660 nm; 120 mW; 5 J/cm^2^; 42 s/point; points 1 cm apart along incision	Standard postoperative care alone	Standard care and pharmacological analgesia upon request	Two sessions: first at 24–30 h after CS, second 24 h later at 48 h postoperative	Immediate postoperative period after CS; enrolled 24–30 h after CS	Postpartum women aged ≥ 18 years, 24–30 h postoperative after CS, mean surgical-wound VAS pain ≥ 3	Pain intensity by VAS and wound-healing quality by Vancouver Scar Scale	48 h postoperatively
Saffarieh et al. 2020 [14]	Iran	65	LLLT using Mustang 2000 device; 830 nm; 4 J/cm^2^; probe at 90° to skin	Sham/imitation laser under the same conditions, but no laser emission	Standard postoperative hospital care	Six sessions on postoperative days 1, 3, 5, 7, 9, and 11	First CS for obstetric reasons; randomized after CS	Age 17–37 years, gestational age 37–42 weeks, first CS, no chronic disease delaying wound repair, no relevant drug use, no prior suprapubic scar, no keloid history, no obstetric complications such as pre-eclampsia/chorioamnionitis/abruption/previa/meconium	Pain at the incision site by VAS and abdominal scar formation by the Vancouver Scar Scale	Pain was assessed on days 5 and 10; scar assessment/photos were followed up to 6 months after surgery
Shweal et al. 2025 [13]	Egypt	50	HLLT using scanner model HPL 3.2; 1 diode 808 nm + 1 diode 915 nm; 1600 mW each diode; 70% power; energy density 5 J/cm^2^; scanner covered incision for 5 min	Medical treatment only	Medical treatment (NSAIDs and antibiotics) for both groups	Three sessions: postoperative days 2, 3, and 4; 5 min/session	Postoperative lower-segment CS; intervention on days 2–4 after CS	Women aged 20–40 years, BMI < 30 kg/m^2^, lower-segment CS	Incisional pain by VAS and serum cortisol level	Post-treatment assessment after 3-session course; effectively day 4 postoperative/after treatment

ASA = American Society of Anesthesiologists; BMI = body mass index; cm = centimeter; CS = cesarean section; GaAlAs = gallium–aluminum–arsenide; h = hour(s); HLLT = high-level laser therapy; HPL = high-power laser; IR = infrared; IV = intravenous; J = joule; J/cm^2^ = joules per square centimeter; kg/m^2^ = kilograms per square meter; LLLT = low-level laser therapy; min = minute(s); mW = milliwatt; nm = nanometer; NR = not reported; NRS = numerical rating scale; NSAIDs = non-steroidal anti-inflammatory drugs; PLP-IR = pulsed light probe-infrared; PLP-R = pulsed light probe-red; s = second(s); VAS = visual analog scale; VSS = Vancouver Scar Scale; W = watt; W/cm^2^ = watts per square centimeter.

**Table 2:** Baseline characteristics of the participants.

Study ID	Number of Patients in Each Group		Age (Years), Mean (SD)		Gestational Age (Weeks), Mean (SD)		BMI, Mean (SD)		Gravidity, Mean (SD)		Parity, Mean (SD)		Smoking, n (%)		Baseline Wound/Pain Status	
	Laser	Control	Laser	Control	Laser	Control	Laser	Control	Laser	Control	Laser	Control	Laser	Control	Laser	Control
Aboelmakarem et al. 2025 [16]	20	20	32.25 ± 5.63	32.40 ± 4.64	NR	NR	29.64 ± 4.11	30.42 ± 3.50	NR	NR	NR	NR	NR	NR	Pre-treatment VAS at first assessment: 9.0 ± 0	Pre-treatment VAS at first assessment: 9.0 ± 0
de Holanda Araujo et al. 2020 [20]	Exp I: 18; Exp II: 19	No-LLLT control: 20; placebo: 20	NR	NR	Exp I: 38.80 ± 2.04; Exp II: 38.47 ± 2.31	Control: 39.00 ± 1.44; Placebo: 39.17 ± 1.47	NR	NR	Exp I: 2.10 ± 1.11; Exp II: 1.79 ± 0.82	Control: 1.63 ± 0.83; placebo: 2.08 ± 1.28	NR	NR	NR	NR	Baseline NRS: Exp I 5.24 ± 2.42; Exp II 5.47 ± 2.00. Pain threshold: Exp I 0.58 ± 0.30; Exp II 0.67 ± 0.33 kgf/cm^2^	Baseline NRS: control 5.75 ± 1.44; placebo 3.90 ± 2.40. Pain threshold: control 0.77 ± 0.47; placebo 0.72 ± 0.54 kgf/cm^2^
Kahkhaie et al. 2023 [17]	120	120	29.78 ± 5.824	27.88 ± 6.038	NR	NR	20.225 ± 1.953	20.817 ± 3.446	Mean NR; first pregnancy 30/120 (25.0%); ≥2 pregnancies 90/120 (75.0%)	Mean NR; first pregnancy 48/120 (40.0%); ≥2 pregnancies 72/120 (60.0%)	NR	NR	4/120 (3.3%)	33/120 (27.5%)	Primary tissue swelling: 33/120 (27.5%)	Primary tissue swelling: 85/120 (70.8%)
Mokmeli et al. 2009 [18]	7	7	27.57 ± 2.70	25.85 ± 2.97	NR	NR	NR	NR	NR	NR	NR	NR	NR	NR	NR	NR
Poursalehan et al. 2018 [19]	40	40	24.62 ± 2.23	25.05 ± 1.78	NR	NR	NR	NR	NR	NR	NR	NR	NR	NR	NR	NR
Quirino de Melo Trindade et al. 2026 [15]	52	52	30 ± 6	30 ± 6	37.9 ± 1.4	37.9 ± 1.8	34.4 ± 6.7	29.9 ± 5.6	2.6 ± 1.9	2.4 ± 1.4	2.1 ± 1.2	2.1 ± 1.2	NR	NR	NR	NR
Saffarieh et al. 2020 [14]	32	33	29.25 ± 2.74	29.90 ± 3.04	NR	NR	27.40 ± 3.09	27.95 ± 3.08	NR	NR	NR	NR	NR	NR	NR	NR
Shweal et al. 2025 [13]	25	25	29.04 ± 2.37	29.08 ± 2.75	NR	NR	24.38 ± 3.10	24.30 ± 2.93	NR	NR	NR	NR	NR	NR	Baseline VAS: 8.66 ± 0.62	Baseline VAS: 8.61 ± 0.66

BMI = body mass index; Exp = experimental group; kgf/cm^2^ = kilogram-force per square centimeter; LLLT = low-level laser therapy; N = number; NR = not reported; NRS = numerical rating scale; SD = standard deviation; VAS = visual analog scale.

**Table 3:** GRADE evidence profile.

Outcomes	Participants (Studies)	Risk of Bias	Inconsistency	Indirectness	Imprecision	Publication Bias	Overall Certainty of Evidence	Effect Estimates
Pain score at 24 h	197 (3 RCTs)	Serious ^a^	not serious	not serious	not serious	not assessed ^e^	⊕⊕⊕◯ Moderate	SMD 0.95 lower (1.35 lower to 0.55 lower)
Pain score at 48 h	181 (2 RCTs)	Serious ^a^	not serious	not serious	not serious	not assessed ^e^	⊕⊕⊕◯ Moderate	SMD 0.71 lower (1.01 lower to 0.41 lower)
Time until first analgesic request	320 (2 RCTs)	Serious ^a^	very serious ^b^	not serious	not serious	not assessed ^e^	⊕◯◯◯ Very low	MD 118.05 min higher (78.37 higher to 157.74 higher)
Total postoperative analgesia dose	320 (2 RCTs)	Serious ^a^	very serious ^b^	not serious	not serious	not assessed ^e^	⊕◯◯◯ Very low	SMD 2.18 lower (3.41 lower to 0.96 lower)
Any analgesia request	181 (2 RCTs)	Serious ^a^	not serious	not serious	serious ^c^	not assessed ^e^	⊕⊕◯◯ Low	RR 0.83 (0.66 to 1.04)
Vancouver Scar Scale	169 (2 RCTs)	Serious ^a^	very serious ^b^	not serious	serious ^c^	not assessed ^e^	⊕◯◯◯ Very low	MD 1.98 lower (4.70 lower to 0.73 higher)
Adverse events	566 (5 RCTs)	Serious ^a^	not serious	not serious	very serious ^d^	not assessed ^e^	⊕◯◯◯ Very low	RR 1.86 (0.37 to 9.43)

CI: confidence interval; MD: mean difference; RCT: randomized controlled trial; RR: risk ratio; SMD: standardized mean difference. ^a^ Downgraded for risk of bias because several contributing trials had some concerns or a high overall risk of bias, mainly related to the randomization process, deviations from intended interventions, outcome measurement, or selection of the reported result. ^b^ Downgraded two levels for very substantial heterogeneity (I^2^ > 75%). ^c^ Downgraded for imprecision because the confidence interval crossed the null and included potentially important benefit and no effect or harm. ^d^ Downgraded two levels for imprecision because events were rare and the confidence interval was very wide, including both appreciable benefit and harm. ^e^ Publication bias was not assessed because fewer than 10 studies contributed to each outcome.

## Data Availability

No new data were created or analyzed in this study. All data are provided in the manuscript and its related Supplementary Materials.

## Supplementary Materials

The following supporting information can be downloaded at https://www.mdpi.com/article/10.3390/healthcare14213647/s1: Table S1: The PRISMA 2020 checklist; Table S2: Search strategy; Table S3: Excluded records during full-text screening.
